# Metabolic Evaluation of Urine from Patients Diagnosed with High Grade (HG) Bladder Cancer by SPME-LC-MS Method

**DOI:** 10.3390/molecules26082194

**Published:** 2021-04-11

**Authors:** Kamil Łuczykowski, Natalia Warmuzińska, Sylwia Operacz, Iga Stryjak, Joanna Bogusiewicz, Julia Jacyna, Renata Wawrzyniak, Wiktoria Struck-Lewicka, Michał J. Markuszewski, Barbara Bojko

**Affiliations:** 1 Department of Pharmacodynamics and Molecular Pharmacology, Faculty of Pharmacy, Collegium Medicum in Bydgoszcz, Nicolaus Copernicus University in Toruń, 85-089 Bydgoszcz, Poland; k.luczykowski@cm.umk.pl (K.Ł.); n.warmuzinska@cm.umk.pl (N.W.); Sylwia_1292@op.pl (S.O.); i.stryjak@cm.umk.pl (I.S.); j.bogusiewicz@cm.umk.pl (J.B.); 2 Department of Biopharmacy and Pharmacodynamics, Faculty of Pharmacy, Medical University of Gdańsk, 80-416 Gdańsk, Poland; julia.jacyna@gumed.edu.pl (J.J.); renata.wawrzyniak@gumed.edu.pl (R.W.); wiktoria.struck-lewicka@gumed.edu.pl (W.S.-L.); michal.markuszewski@gumed.edu.pl (M.J.M.)

**Keywords:** bladder cancer (BC), metabolomics, solid phase microextraction (SPME), liquid chromatography, mass spectrometry, urine

## Abstract

Bladder cancer (BC) is a common malignancy of the urinary system and a leading cause of death worldwide. In this work, untargeted metabolomic profiling of biological fluids is presented as a non-invasive tool for bladder cancer biomarker discovery as a first step towards developing superior methods for detection, treatment, and prevention well as to further our current understanding of this disease. In this study, urine samples from 24 healthy volunteers and 24 BC patients were subjected to metabolomic profiling using high throughput solid-phase microextraction (SPME) in thin-film format and reversed-phase high-performance liquid chromatography coupled with a Q Exactive Focus Orbitrap mass spectrometer. The chemometric analysis enabled the selection of metabolites contributing to the observed separation of BC patients from the control group. Relevant differences were demonstrated for phenylalanine metabolism compounds, i.e., benzoic acid, hippuric acid, and 4-hydroxycinnamic acid. Furthermore, compounds involved in the metabolism of histidine, beta-alanine, and glycerophospholipids were also identified. Thin-film SPME can be efficiently used as an alternative approach to other traditional urine sample preparation methods, demonstrating the SPME technique as a simple and efficient tool for urinary metabolomics research. Moreover, this study’s results may support a better understanding of bladder cancer development and progression mechanisms.

## 1. Introduction

Bladder cancer (BC) is mainly diagnosed among people over 50 years of age. It is diagnosed three times more often in men and represents the fourth most common cancer in this group. Approximately 90% of bladder cancers are transitional cell carcinoma, while the other 10% are squamous cell carcinoma and adenocarcinoma [1,2]. Among the recognized risk factors—contributing to the progress of neoplastic changes in the bladder—smoking is the most significant [3]. Active smokers have a four times higher risk of developing the disease compared to persons who have never smoked. Other causes include genetic disorders, exposure to specific chemical compounds, or chronic bladder irritation [2]. Hematuria is the most common symptom of bladder cancer. Other possible symptoms include weight loss and pain in the abdomen and in the kidneys. However, these symptoms are not specific to bladder cancer, making diagnosis challenging since it requires exclusion of other diseases [2]. Commonly performed tests for cases where there is a suspicion of bladder cancer include cystoscopy (the “gold standard”), cytology of urinary sediment, and imaging diagnostics. However, these diagnostic methods have significant drawbacks: cystoscopy is invasive and uncomfortable for the patient, whereas laboratory tests have low sensitivity and specificity for early stages of bladder cancer [1]. These limitations have fostered research on alternative, more effective, minimally invasive approaches for bladder cancer diagnosis. As a first step in the creation of new diagnostic tools, untargeted metabolomics/metabolomic profiling of biological fluids can be used to identify biomarkers of bladder cancer [4], opening new paths towards the creation of novel biomarker-based diagnostic tools.

Human urine is a biological matrix that is easy to obtain in large volumes. In addition to its easy availability, urine is an important biological matrix due to the type of information it may hold in relation to the various metabolic processes occurring throughout the human body. Urine is produced by the kidneys, which filter unwanted substances from the body; as such, urine contains many unnecessary and harmful by-products of metabolism. The main components of urine are water, urea, creatinine, ammonia, inorganic salts, and other water-soluble substances [5]. Given that urine is largely free from interfering lipids or proteins, sample preparation for this matrix is significantly less time-consuming in comparison to other biological matrices. Hence, metabolomic studies using urine as a biological matrix are promising [5]. Metabolites present in the urine, which are the final products of processes occurring in healthy and cancerous cells, can be of particular importance in the diagnosis of bladder cancer. Differences observed between metabolomic profiles of BC patients compared to healthy persons may allow us to find potential BC indicators [6].

Even though urine is a relatively simple biological matrix, it still requires careful sample preparation considerations with respect to issues such as matrix effects and sample-to-sample variations. The ideal sample preparation method should be simple (with the smallest number of steps) and reproducible. Besides, it must ensure sample purification, recovery of a wide range of analytes, as well as inhibition of metabolism, which protects compounds from degradation [7]. The simplest and most common preparation method of urine samples prior to MS analysis is dilution (“dilute and shoot”). However, this simplistic strategy compromises sensitivity and does not ensure metabolism quenching, thus spurring the development of alternative approaches to urine sample preparation to address these shortcomings. Of the variety of methods developed for urine analysis, solid-phase microextraction has been demonstrated to largely fulfill the abovementioned conditions, given that in addition to integrating various steps of the sample preparation and extraction process, it additionally provides efficient sample clean-up, making it compatible with liquid chromatography and mass spectrometry [7,8]. In addition, the technology offers flexibility in terms of device geometry, chemistry of the extraction phase, and high-throughput capabilities. With regards to the latter, the most common format of the device i.e., the microfiber, is widely used for low-throughput analysis of tissues or in animal studies [9,10], as well as for high throughput analyses where sample volumes are limited [11]. When sample volume is of no concern, the thin film format of SPME (TFME) is preferable, as it increases recovery of analytes and is compatible with commercial automated or semi-automated high throughput robotic systems [10].

In the current study, thin film SPME in high throughput semi-automated mode was used for metabolomics screening of urine of patients with bladder cancer and healthy controls. Following experimental data acquisition, statistical analysis, and a biological pathway analysis were carried out to identify compounds that might be important in the identification of BC as well as in the identification of metabolic pathways involved in the pathogenesis and progression of the disease.

## 2. Results

### 2.1. Subject’s Characteristics

Urine samples used for the study were obtained from advanced-stage bladder cancer patients and from healthy volunteers. Both groups consisted of 24 patients at a similar age, with similar BMI values, and with a similar number of smokers. The average age of patients in both groups fell within the age bracket known for carrying the highest risk of developing bladder cancer. A summary of patient demographics for the two groups is presented in Table 1. The group of healthy volunteers did not undergo any treatment during the sample collection period, and they were declared to be in good health condition, which was also confirmed with laboratory results. The enrollment of BC patients depended on confirmation of high grade, muscle-invasive BC during the histopathological evaluation of biopsies collected during the diagnostic procedure.

### 2.2. Untargeted Metabolomics Analysis

**Figure 1:**
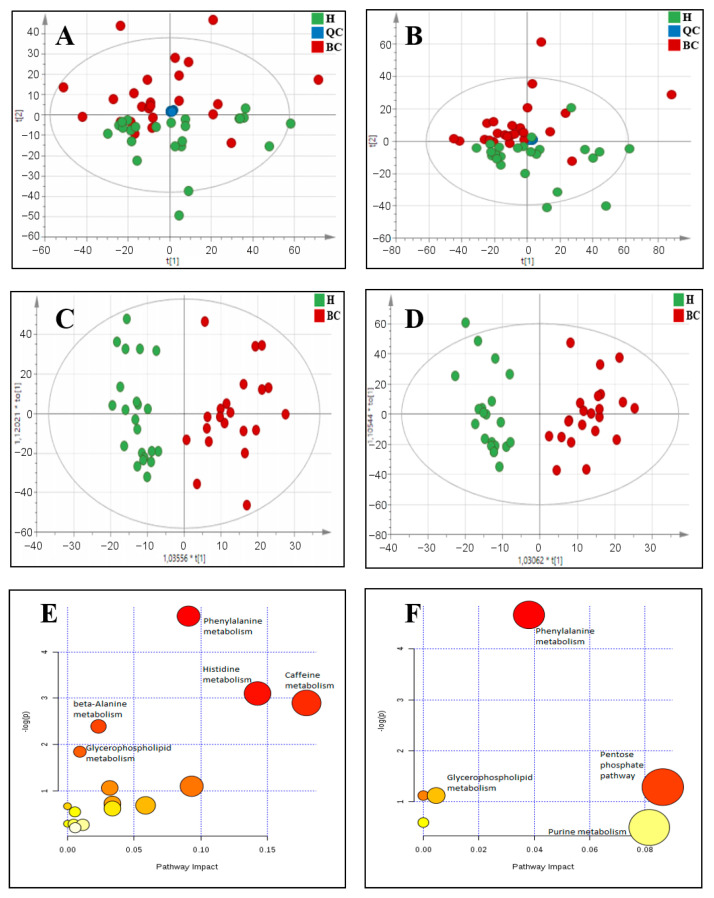
Observed differences in metabolic profiles of control and BC patients: (**A**) PCA score plot for positive ionization mode, with PC1 describing 12.8% of the variation and PC2 describing 5.5%, (**B**) PCA plot for negative ionization mode, with PC1 describing 13.6% of the variation and PC2 describing 5.5%, (**C**) Score plot of OPLS-DA model for positive ionization mode, with R2 = 85.2% and Q2 = 46%, (**D**) Score plot of OPLS-DA model for negative ionization mode, with R2 = 88.2% and Q2 = 43.3%, (**E**) Pathway analysis of differential metabolites in positive ionization mode, (**F**) Pathway analysis of differential metabolites in negative ionization mode.

The attained data was subjected to principal component analysis (PCA) in order to assess data quality as well as determine differences between the metabolic profiles of control and BC patients. As shown in Figure 1A,B, QC samples formed a tight cluster, confirming the quality of the obtained results. Additionally, the two studied groups achieved relatively good separation in both positive and negative ionization modes. Based on the PCA score plots and a 95% confidence ellipse using Hotelling T-squared, five outliers were removed: three from the group of BC patients and two from the healthy group for positive ionization mode, and two from the group of BC patients and three from the healthy group for negative ionization mode. Thereafter, supervised multivariate analysis: orthogonal projections to latent structures discriminant analysis (OPLS-DA) was performed to achieve maximum separation among the groups (Figure 1C,D). The Q2 and R2 values for the model were 46% and 85.2% for positive ionization mode and 43.3% and 88.2% for negative ionization mode, respectively.

Based on the obtained model, metabolites found to contribute the most in differentiating the two groups according to their VIP scores were selected for further analysis. A VIP score value >1 was selected as cut-off value. Table 2 shows the metabolites meeting the abovementioned criteria. Additional information regarding the annotation of these compounds is presented in Supplementary Information Table S1.

**Figure 2:**
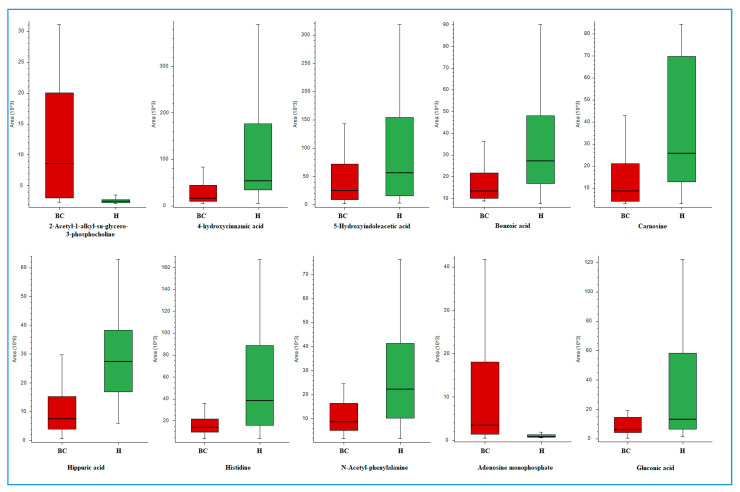
Box Whisker charts for selected compounds differentiating the studied groups (red (BC)—bladder cancer patients, green (H)—healthy volunteers).

Figure 2 presents Box Whisker plots for selected annotated metabolites exhibiting significant differences in urinary levels between BC patients and healthy controls. Chromatograms for each of these compounds are shown in Supplementary Information Figure S2. Box Whisker plots represent peak areas for a selected component on the y axis as a rectangle against equally spaced sample groups on the x axis. The height of the rectangle represents the peak areas in the interquartile range. The following equations were used to calculate the upper and lower whiskers: Interquartile range (IQR) = Quartile 3 (Q3) − Quartile 1 (Q1), Upper whisker = Q3 + IQR × 1.5, Lower whisker = Q1 − IQR × 1.5.

### 2.3. Pathway Analysis

A pathway analysis was performed to obtain biological information about relevant networks of metabolic pathways that undergo changes in patients with bladder cancer in comparison to the healthy population. Our pathway analysis was carried out using the list of significantly differential metabolic features previously determined as described above (VIP score > 1). Results obtained from both ionization modes are presented in Figure 1E,F. The attained results indicate that the identified compounds may be mainly associated with phenylalanine, histidine, and caffeine metabolism. Table 3 shows the full list of identified metabolites and associated pathways in both positive and negative ionization modes.

## 3. Discussion

In recent years, the urology field has seen rapid development in terms of urine-based metabolomic analysis. Given that urine comes in direct contact with bladder epithelial cells and tumor tissue, it may therefore contain metabolites shed from cancerous cells in the bladder [6]. In metabolomics, sample preparation has appreciable influence on the quality of the obtained results, particularly for biofluid samples such as urine [18]. In the current study, a technology integrating sample preparation and extraction of metabolites, namely solid phase microextraction, was presented for metabolomics analysis of urine samples of bladder cancer patients. It has been already demonstrated that SPME is an effective tool for metabolomics studies in plasma and tissue [8,10], but no direct immersion urinary metabolomics analyses have been reported to date to the best of our knowledge. However, extensive anti-doping multi-residue analyses in urine have been successfully carried out via the thin film SPME automated high throughput system [19], showing the potential of the technology for simultaneous determination of over 100 compounds with no matrix effect and good sensitivity in urine, all while affording a simple analytical protocol. Herein, thin film SPME followed by LC-HRMS analysis was used for the first time for urinary untargeted metabolomics in cancer patients.

The metabolites selected as discriminant compounds and, subsequently, the results of the pathways analysis, are consistent with previous reports [13]. Compounds found present at decreased levels in BC patients in comparison to healthy controls included benzoic acid (BA) and hippuric acid (HA), which are known to be involved in the phenylalanine metabolism pathway. Hippuric acid is a normal component of urine as a metabolite of aromatic compounds from food [20]. While the serum concentration of HA is increased in uremic patients as a result of reduced renal clearance [21], lower levels of hippuric acid in urine samples from BC patients are in agreement with previous studies, making it characteristic for this group of patients [14,15]. Benzoic acid, a compound commonly used as a food preservative, conjugates to glycine in the liver and is excreted as hippuric acid [20]. Benzoic acid and hippuric acid are also believed to play essential roles in gut microbial pathways [14,22]. The important role of gut microbiota in health has been gradually gaining recognition in science; although studies regarding the physiological functions of BA have mainly involved pigs as a research model for humans [22], benzoic acid administration has been found to improve gut functions including digestion and absorption, and also could improve gut barrier function, including nonspecific barrier mechanisms and specific immunological responses [22]. The lower concentrations of hippuric acid and benzoic acid found in the bladder cancer group might be associated with gut microbial pathways, decreased activity of the hippurate hydrolase, or increased activity of the glycine *N*-benzoyltransferase [14].

Another down-regulated metabolite was *N*-Acetyl-phenylalanine, which also participates in the phenylalanine metabolism pathway. Acetylphenylalanine is a product of enzyme phenylalanine *N*-acetyltransferase [20,23], and lower concentrations of this compound in saliva have been previously reported in oral squamous cell carcinoma patients [23]. Histidine and carnosine also exhibited decreased levels in BC patients. The latter plays a role in histidine, beta-alanine, and nitrogen metabolism as well as in the aminoacyl-tRNA biosynthesis pathway. Histidine is an α-amino acid that is used in the biosynthesis of proteins. One of the proteins significantly related to tumor progression is fragile histidine triad protein (FHIT), a protein that has been proven to have suppressor properties. The lack of FHIT protein or its reduced level is observed in many types of cancer as well as in various cancer cell lines [24,25,26]. Reduced concentration of histidine in bladder cancer patients may correlate with a lower activity of FHIT protein [24]. FHIT protein deficiency has been also reported in bladder cancer, mainly in high-grade tumors. This phenomenon correlates with poorer prognosis in BC patients [26,27,28]. Histidine is also a precursor for carnosine biosynthesis. This dipeptide links histidine and the beta-alanine metabolism pathway. While carnosine has not been reported in the literature as a metabolite of significant importance in bladder cancer, numerous studies have shown that carnosine exhibits some antioxidant effects [29,30,31]. Carnosine’s ability to scavenge reactive oxygen species (ROS) may be important in the prevention of cancers. In this study, carnosine levels were found to be higher in urine of the control group, which would be in line with these assumptions.

The 2-Acetyl-1-alkyl-sn-glycero-3-phosphocholine levels in urine exhibited relevant differences in comparisons of metabolomic profiles of bladder cancer patients and healthy volunteers. This compound is also known as a platelet-activating factor (PAF), playing a role in platelet aggregation, inflammation, and allergic reactions. PAF and its receptor have been implicated in malignant processes such as tumor development, growth, and metastatic angiogenesis [32]. Kispert et al. observed significant increases in PAF accumulation in bladder cancer cells following cigarette smoke extract exposure [12]. The increased levels of PAF in BC patients are in accordance with the current study; however, the number of smokers in both studied groups was similar, which might suggest that smoking is not the only factor influencing PAF accumulation in the bladder.

A higher level of 4-hydroxycinnamic acid was found in the urine of healthy volunteers compared to patients with bladder cancer. This organic compound, also known as *p*-Coumaric acid, is a naturally occurring phenolic acid present in most plants, including commonly consumed vegetables and fruits [33]. There are numerous reports describing its antioxidant and anticancer properties [34,35,36]. Kong et al. have shown that *p*-coumaric acid inhibits tumor growth through reduction of angiogenesis within the tumor [36]. While our current findings cannot directly establish a relationship between 4-hydroxycinnamic acid and bladder cancer, the higher levels of this compound in healthy volunteers may be evidence of its role in cancer prevention.

We have also identified changes in the pentose phosphate pathway demonstrated by the observed decrease in gluconic acid levels. Gluconic acid, a naturally occurring carboxylic acid, as well as its derivatives are used in food, pharmaceuticals, and cosmetics as additives or buffer salts; however, the mechanisms of their biological activity are still not completely understood [20,37]. Recent research in this area has indicated that gluconic acid and its derivatives may contain antioxidative properties [37]. Lower concentrations of this compound in the BC group as compared to the healthy control might be related to oxidative stress, which has been implicated in the pathogenesis of numerous diseases [38]. Decreased concentration of gluconic acid in the urine of the bladder cancer patients has been reported previously [16,17]. Interestingly, gluconic acid such as carnosine and *p*-coumaric acid has antioxidative properties and was also found to be present in lower quantities in the urine of the BC group.

We acknowledge that there are limitations to our study. The analysis was performed with a relatively small number of patients without differentiation of cancer type. However, as emphasized throughout the manuscript, the results do not provide a well-validated panel of biomarkers mainly due to the small number of participants and larger cohort study to confirm or deny this work’s results. On the other hand, based on this study, we were able to identify some interesting metabolites not described previously in the view of BC that might contribute to the pathogenesis of bladder cancer or serve as protective compounds in cancer development, and this information can be a good starting point for targeted analysis of these compounds and the pathway they are involved in. Moreover, our work has demonstrated that TFME hyphenated to LC-HRMS can be an alternative platform for untargeted urinary analysis toward biomarker discovery.

## 4. Materials and Methods

Analytical grade sodium chloride, potassium chloride, potassium phosphate monobasic, sodium phosphate dibasic, hydrochloric acid, and sodium hydroxide, used for the phosphate-buffered saline solution (PBS pH 7.4) preparation, as well as the LC-MS grade chromatographic solvents water, methanol, acetonitrile, and formic acid, were purchased form Sigma-Aldrich (Poznań, Poland).

Urine was obtained from advanced-stage bladder cancer patients and from healthy volunteers (control group). The urine samples were collected from the first urination in the morning. Samples were provided by the Department of Biopharmaceutics and Pharmacodynamics, Medical University of Gdańsk.

**Figure 3:**
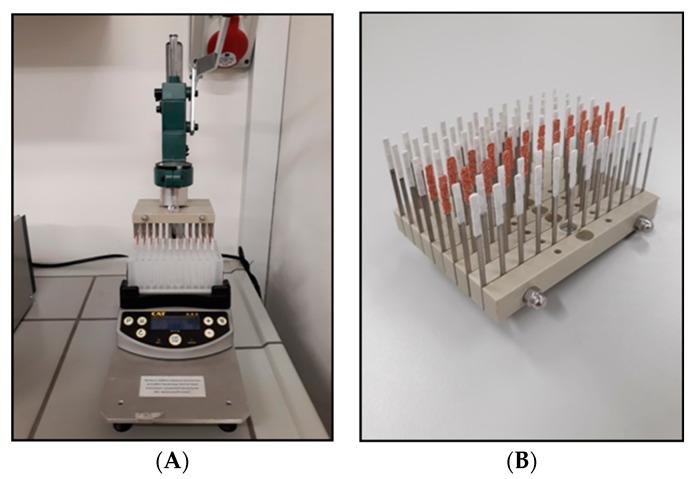
The high throughput 96-semi-automated SPME system used for analysis of urine samples (**A**) and a set of 96 SPME devices coated with different types of extraction phases (the PS-DVB coating selected for the study is represented in rows 3 and 4 from the right) (**B**).

Solid phase microextraction (SPME) in its thin film format was used for preparation of samples, and each step of the process was carried out on a high throughput 96-semi-automated SPME system (Professional Analytical System (PAS) Technology, Magdala, Germany), which allowed for simultaneous analysis of all samples (Figure 3). Extraction was performed by using steel blades coated with a polystyrene divinylbenzene (PS-DVB) sorbent (Alchem, Toruń, Poland). Coating preparation procedures were based on the spraying method described by Mirnaghi et al. [39]. Steel blades were purchased form Professional Analytical System (PAS) Technology (Magdala, Germany) and polypropylene Nunc 96 DeepWell plates were purchased from Sigma-Aldrich (Poznań, Poland).

Prior to the analysis, urine was diluted in PBS (1:1; *v*/*v*) so as to establish a matrix pH of 7.4. Before extraction, coatings were conditioned for 30 min with 1.0 mL of a methanol: water (1:1; *v*/*v*) solution in 96-well-plates with agitation set at 750 rpm to improve sorbent surface activation and prepare the sorbent to retain the analytes, then subsequently submitted to a 10 s wash step to remove residual methanol. The extraction process was executed from 0.5 mL of urine samples for 1 h at 37 °C. After extraction, the blades were placed in 1 mL of nanopure water for 10 s to remove particulates, salts, and other contaminants loosely attached on the coating surface by non-specific interactions, which could potentially cause matrix effects and instrument contamination. Following this wash step, desorption was conducted in 1 mL of acetonitrile: water (1:1; *v*/*v*) solution with agitation (750 rpm) for 90 min.

### 4.1. LC-MS Conditions

Chromatographic separation was performed on the Dionex UHPLC system. Urine extracts, obtained as described above, were injected at a volume of 10 µL on a reversed phase pentafluorophenyl (PFP) column (Discovery HS F5 100 mm × 2.1 mm, 3 μm (Sigma-Aldrich, Poznań, Poland)). Autosampler and column temperatures were set to 4 °C and 25 °C, respectively. The flow rate was 0.3 mL/min. The mobile phase A was water with formic acid (99.9:0.1; *v*/*v*) and mobile phase B was acetonitrile with formic acid (99.9:0.1; *v*/*v*). The total time of analysis per sample was 40 min. The starting mobile phase conditions were 0% B from 0 to 3.0 min, followed by a linear gradient to 90% B from 3.0 to 25.0 min, an isocratic hold at 90% B until 34.0 min, and a 6 min column re-equilibration time [40]. Total ion chromatograms of QC samples in both ionization modes are shown in Supplementary Information Figure S1.

The analyses were performed in both positive and negative electrospray ionization modes in separate runs on a Q Exactive Focus Orbitrap mass spectrometer (Thermo Fisher Scientific). In positive ionization mode, the following HESI ion source parameters were used: spray voltage 1500 V, capillary temperature 300 °C, sheath gas 40 a.u., aux gas flow rate 15 a.u., probe heater temperature 300 °C, and S-Lens RF level 55%. For negative ionization mode, HESI ion source parameters were as follows: spray voltage 2500 V, capillary temperature 256 °C, sheath gas 48 a.u., aux gas flow rate 11 a.u., probe heater temperature 413 °C, and S-Lens RF level 55%. Scan range was set on *m*/*z* 80–1000 with resolution 70,000. The instrument was calibrated using external calibration immediately before the analysis and every 48 h, resulting in mass accuracy <2 ppm. Data acquisition was performed with Xcalibur software v. 4.0.

All samples were analyzed in one randomized sequence and QC samples were run periodically (8–10 injections) to verify instrument performance. QC samples were prepared by mixing 20 μL of each of the 48 urine extracts.

The putative identification of compounds was confirmed based on Full MS/dd-MS2 mode. Fragmentation parameters were as follows. Mass resolution: 35,000 full width at half maximum (FWHM), AGC target: 2E4, minimum AGC: 8E3, intensity threshold: auto, maximum IT: auto, isolation window: 3.0 *m*/*z*, stepped collision energy: 10 V, 20 V, 40 V, loop count: 2, dynamic exclusion: auto. Fragmentation spectra were confirmed with online databases such as LIPID MAPS, HMDB, METLIN, and mzCloud.

### 4.2. Data Processing and Statistical Analysis

Raw data was processed by the Compound Discoverer 3.0 (Thermo Fisher Scientific) software with aims to identify metabolites present in the samples. Detected metabolites with signal-to-noise >3 and peak intensity >1,000,000 were subjected to analysis. Intensity tolerance was set as 30%, and RT tolerance as 0.2 min. QC-based area was used for correction (min 50% coverage, max 30% RSD in QC, normalization by constant mean). A data filtration step removed 49% of the 7907 features and 29% of the 6114 features in positive and negative ionization modes, respectively. After peak alignment, gap filling was applied to fill missing values by a very small peak at the level of spectrum noise for the compound. The obtained table with accurate masses of annotated compounds was inserted into SIMCA 15 (Umetrics), where principal component analysis (PCA) and orthogonal projections to latent structures discriminant analysis (OPLS-DA) were performed using unit variance (UV) scaling. Statistically significant compounds in the projection used in OPLS-DA models were selected using Variable Importance in Projection (VIP) scores >1 and then filtered, taking into account the biological relevance and consistency of the fragmentation spectrum with databases. Pathway analysis was carried out with MetaboAnalyst 4.0, using Homo sapiens Kyoto Encyclopedia of Genes and Genomes (KEGG) metabolic pathway database. The analysis used the hypergeometric test as an enrichment method and relative-betweenness centrality for topology analysis, and the results were presented in the form of scatter plots.

## 5. Conclusions

The main goals of this study were to test applicability of TFME for untargeted screening of urinary metabolites and the identification of compounds involved in tumor development and progression in bladder cancer patients. Multivariate OPLS-DA analysis was performed to differentiate BC patients from healthy individuals. This profiling has not only identified a group of metabolites that may contribute to bladder cancer development, but also compounds that show potential in the prevention of cancer. Pathway analysis allowed for the integration of metabolomics data and biological information to enhance knowledge about the biological links between selected metabolites and BC pathogenesis. Future quantitative targeted studies on a larger number of patients are required to validate current findings and to evaluate the predictive value of the selected metabolites. Moreover, the results of the present study demonstrated that thin film SPME can be efficiently used as an alternative approach to other traditional urine sample preparation methods, demonstrating the SPME technique as a simple and efficient tool for urinary metabolomics research.

## Figures and Tables

**Table 1:** Demographic characteristics of the studied groups.

Studied Group	Group Size		Age [years]	BMI [kg/m^2^]	Smokers [%]
	Men	Women			
BC patients	18	6	65 (±12.0)	26.03 (±4.1)	67
Healthy Volunteers	18	6	64 (±10.4)	25.87 (±2.2)	75

**Table 2:** Differential metabolites in positive and negative ionization mode. References indicate BC’s previous clinical studies in which the metabolite was selected as discriminant and/or dysregulated (MW—molecular weight; ↓ indicates down-regulation and ↑ indicates up-regulation in BC).

Metabolites	MW	RT	VIP Score	Trend
**Positive ionization mode**				
2-Acetyl-1-alkyl-sn-glycero-3-phosphocholine [12]	523.3638	17.98	1.66212	↑
3-Dehydroxycarnitine	145.1103	3.51	1.13186	↓
3-Methylxanthine [13]	166.0491	1.38	1.80968	↓
4-Hydroxycinnamic acid	164.0475	4.17	1.80279	↓
5-Hydroxyindoleacetic acid [13]	191.0582	8.42	1.17858	↓
Adenine	135.0545	3.08	2.03098	↑
Benzoic acid [14]	122.0370	4.17	1.38913	↓
Carnosine	226.1064	3.95	1.06014	↓
Epinephrine	183.0896	8.35	1.52508	↓
Hippuric acid [13,14,15]	179.0582	7.83	2.37849	↓
Histidine	155.0695	2.14	1.30363	↓
Isoniazid	137.0589	7.97	1.43638	↓
LysoPE(18:1)	479.3014	17.55	1.66856	↑
*N*-Acetyl-phenylalanine	207.0896	9.17	1.73877	↓
*p*-Aminobenzoic acid [13]	137.0477	1.44	1.45154	↓
Retinol	286.2295	12.93	1.07027	↓
Theophylline	180.0648	6.76	2.21588	↓
**Negative ionization mode**				
3-(3-sulfooxyphenyl)propanoic acid	246.0195	7.18	1.72228	↓
Adenosine monophosphate *	347.0631	1.18	1.60799	↑
Gluconic acid [16,17]	196.0587	1.39	1.74004	↓
Hippuric acid [13,14,15]	179.0583	7.81	2.21443	↓
Indolelactic acid [13]	205.0739	11.00	1.31510	↓

* Fragmentation spectrum not confirmed with online databases.

**Table 3:** Metabolic pathways and associated metabolites in positive and negative ionization mode.

Pathway Name	Metabolites
**Positive Ionization Mode**	
Phenylalanine metabolism	Benzoic acid; Hippuric acid; 4-Hydroxycinnamic acid; *N*-Acetyl-phenylalanine
Histidine metabolism	Histidine; Carnosine;
Caffeine metabolism	Theophylline; 3-Methylxanthine;
beta-Alanine metabolism	Carnosine; Histidine
Glycerophospholipid metabolism	LysoPE(18:1); Phosphatidyl-*N*-dimethylethanolamine;
Retinol metabolism	Retinol;
Ether lipid metabolism	2-Acetyl-1-alkyl-sn-glycero-3-phosphocholine;
Ubiquinone and other terpenoid-quinone biosynthesis	4-Hydroxycinnamic acid;
Drug metabolism—other enzymes	Isoniazid;
Nitrogen metabolism	Histidine;
Folate biosynthesis	*p*-Aminobenzoic acid
Aminoacyl-tRNA biosynthesis	Histidine
Tyrosine metabolism	Epinephrine;
Tryptophan metabolism	5-Hydroxyindoleacetic acid
Purine metabolism	Adenine;
**Negative Ionization Mode**	
Phenylalanine metabolism	Hippuric acid;
Pentose phosphate pathway	Gluconic acid;
Nitrogen metabolism	Adenosine monophosphate;
Glycerophospholipid metabolism	Phosphatidyl-*N*-dimethylethanolamine;
Tryptophan metabolism	Indolelactic acid;
Purine metabolism	Adenosine monophosphate;

## Data Availability

Not applicable.

## Supplementary Materials

The following are available online. Figure S1: Total ion chromatogram of QC sample in positive (A) and negative (B) ionization mode. Figure S2: Chromatograms for selected compounds differentiating the studied groups: 2-Acetyl-1-alkyl-sn-glycero-3-phosphocholine (A), 4-Hydroxycinnamic acid (B), 5-Hydroxyindoleacetic acid (C), Adenosine monophosphate (D), Benzoic acid (E), Hippuric acid (F), Gluconic acid (G), Carnosine (H), Histidine (I), *N*-Acetyl-phenylalanine (J), Table S1: Differential metabolites in positive and negative ionization mode with information on the annotation in the ChemSpider database.

## Sample Availability

Extracts of the urine samples are available from the authors.
