# Supplementary material for: Metabolic Evaluation of Urine from Patients Diagnosed with High Grade (HG) Bladder Cancer by SPME-LC-MS Method
Source: Molecules. 2021 Apr 11;26(8):2194. doi: 10.3390/molecules26082194 (PMC8068997; doi:10.3390/molecules26082194)
Supplement: Supplementary file 1 [file molecules-26-02194-s001.pdf]

Supplementary information

Tab. S1. Differential metabolites in positive and negative ionization mode with information on the annotation in the ChemSpider database (Sfit – Spectral similarity score between theoretical and measured isotope pattern in %)

**Metabolites**	**# ChemSpider results**	**Full match**	**Δ mass [ppm]**	**# matched /missed isotopes**	**Sfit [%]**
**Positive ionization mode**					
Benzoic acid	33	1	1.27	2/0	91
Hippuric acid	345	1	0.30	1/0	100
4-Hydroxycinnamic acid	195	1	0.61	2/0	93
N-Acetyl-phenylalanine	940	1	0.57	2/0	91
Histidine	174	1	0.32	5/0	77
Carnosine	106	1	0.67	2/0	74
Theophylline	93	1	0.08	2/0	83
3-Methylxanthine	71	1	0.10	3/0	85
LysoPE(18:1)	37	1	0.48	3/0	80
Retinol	224	9 (isomers)	0.66	3/0	71
2-Acetyl-1-alkyl-sn-glycero-3-phosphocholine	23	3	0.05	3/0	81
Isoniazid	82	1	0.13	1/0	100
p-Aminobenzoic acid	130	1	0.52	2/0	79
3-Dehydroxycarnitine	295	1	0.04	4/0	79
Epinephrine	365	1	0.51	2/0	86
5-Hydroxyindoleacetic acid	364	1	0.28	2/0	76
Adenine	50	1	0.38	2/0	92
**Negative ionization mode**					
3-(3-sulfooxyphenyl)propanoic acid	8	1	1.14	3/0	92
Hippuric acid	345	1	0.46	3/0	70
Gluconic acid	22	17	2.25	3/0	75
Adenosine monophosphate	56	10	0.10	3/0	73
Indolelactic acid	695	1	0.36	3/0	79

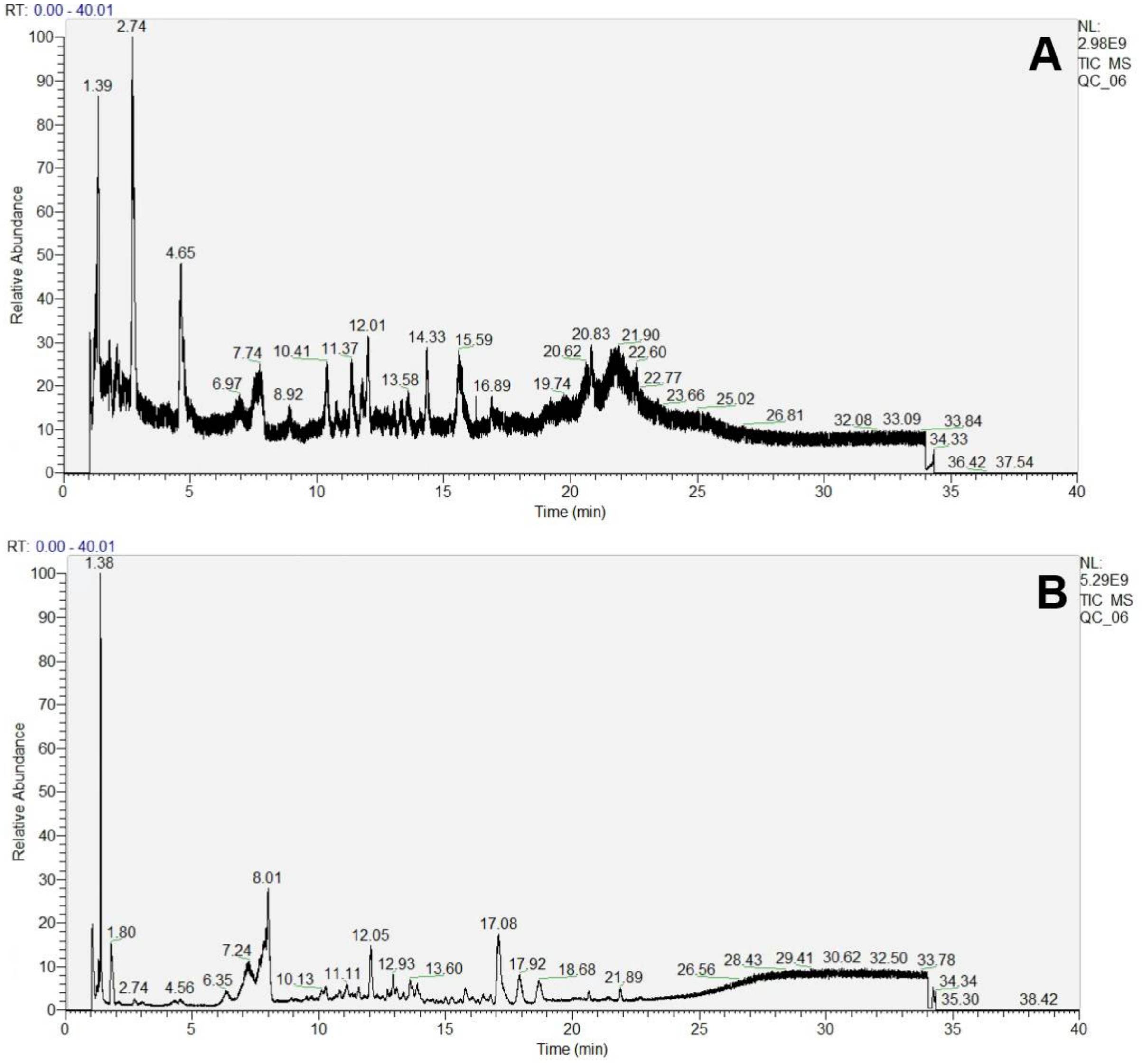

Fig. S1. Total ion chromatogram of QC sample in positive (A) and negative (B) ionization mode.

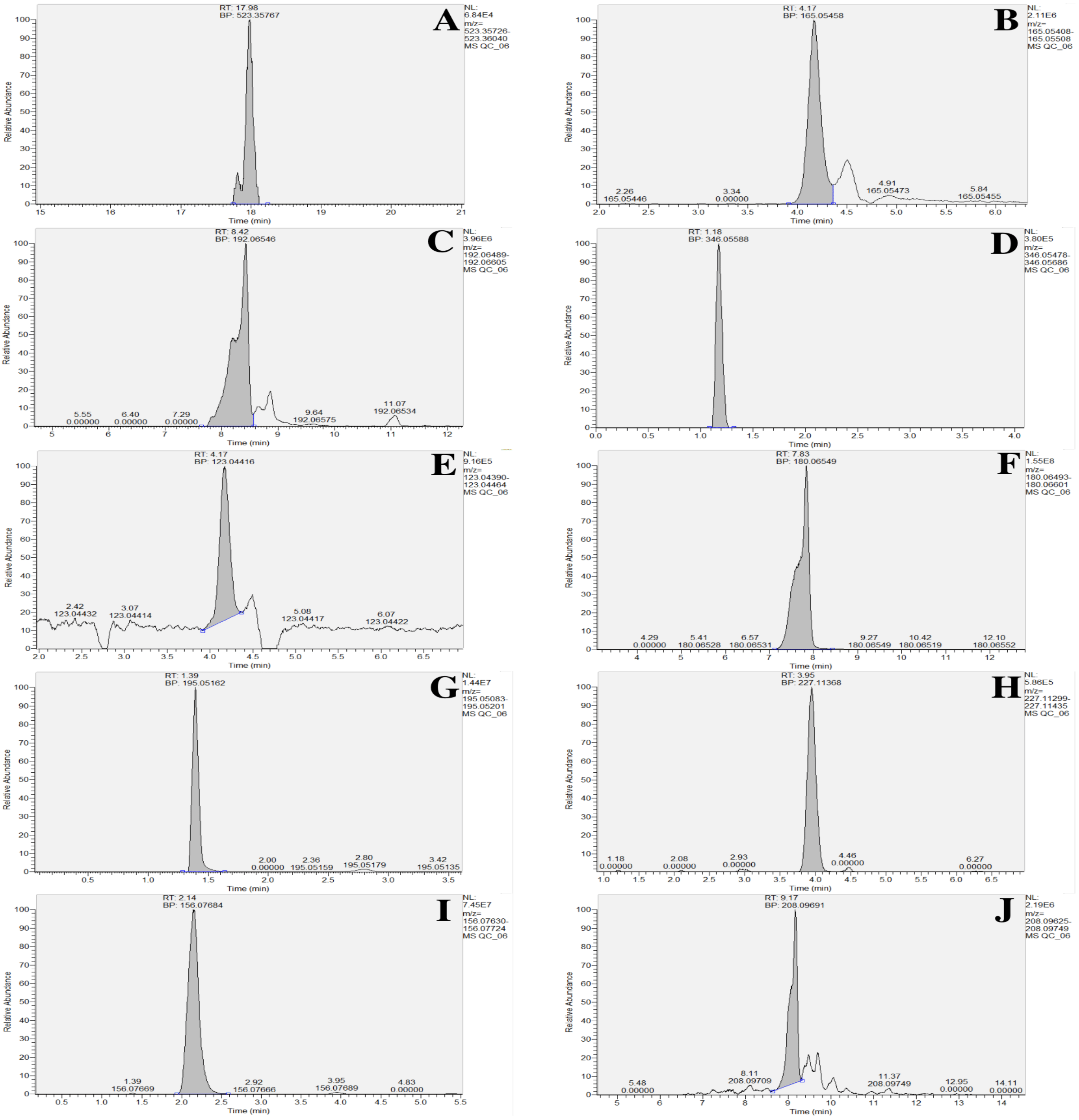

Fig. S2. Chromatograms for selected compounds differentiating the studied groups: 2-Acetyl-1-alkyl-sn-glycero-3-phosphocholine (A), 4-Hydroxycinnamic acid (B), 5-Hydroxyindoleacetic acid (C), Adenosine monophosphate (D), Benzoic acid (E), Hippuric acid (F), Gluconic acid (G), Carnosine (H), Histidine (I), N-Acetyl-phenylalanine (J)
